# Day‐Case Laparoscopic Hysterectomy: A Successful Pilot in Regional New Zealand

**DOI:** 10.1111/ajo.70049

**Published:** 2025-05-28

**Authors:** Ben McLaughlin, Jonathan Panckhurst, Richard Carpenter

**Affiliations:** ^1^ Department of Women's Health Christchurch Women's Hospital, Waitaha Canterbury District, Health New Zealand—Te Whatu Ora Christchurch New Zealand; ^2^ Department of Anaesthesia Nelson Public Hospital, Nelson‐Marlborough District, Health New Zealand—Te Whatu Ora Nelson New Zealand; ^3^ Department of Women's Health Nelson Public Hospital, Nelson‐Marlborough District, Health New Zealand—Te Whatu Ora Nelson New Zealand; ^4^ Department of Obstetrics and Gynaecology The University of Auckland Auckland New Zealand

**Keywords:** day surgery, day‐case, gynaecology, health economics, hysterectomy, laparoscopy

## Abstract

We conducted a day‐case total laparoscopic hysterectomy service improvement project at Nelson Public Hospital, New Zealand, in August and September, 2024. A retrospective analysis was performed, and a voluntary patient questionnaire administered, at the 4‐week postoperative review. Five participants were recruited and successfully discharged on the day of surgery without complication, and either agreed or strongly agreed with the statement, ‘I would recommend day‐case total laparoscopic hysterectomy to friends and family’. Qualitative feedback was strongly positive. We conclude that with appropriate infrastructure and resourcing, offering day‐case total laparoscopic hysterectomy in a regional centre is both viable and acceptable to patients.

## 1 Introduction

Elective surgeries in New Zealand public hospitals are often cancelled due to a lack of inpatient beds; particular pressure is felt during times of high acuity, such as the winter viral season. This situation is further exacerbated by the current climate of staff shortages and financial constraints [1]. It is within this overall context that we have conducted a trial of same‐day discharge, laparoscopic hysterectomy (DCTLH) at Nelson Public Hospital, Nelson‐Marlborough District, Health New Zealand—Te Whatu Ora, a regional hospital at the top of New Zealand's South Island.

Although a novel approach in New Zealand, DCTLH is becoming increasingly common in international centres, at the same time that major operations as a whole are moving toward day‐case approaches [2]. A recent quality improvement project in Sussex, England, showed that DCTLH could be implemented with high patient acceptability rates, no significant complications, and no requirements for additional equipment. Same day discharge was achieved in 93%–100% of patients, and 90% of patients in the cohort reported that they would recommend a day‐case hysterectomy to their friends and family [3]. A smaller study conducted in 2022 at a tertiary hospital at Queensland, Australia found similar results [4]. In New Zealand however, the most common approach encountered by the authors after laparoscopic hysterectomy has been a 1–2 night hospital stay.

## 2 Materials and Methods

This study originated as a service improvement project inspired by the aforementioned work of Ward et al. [3] Its overarching philosophy was that success depends on the whole health ecosystem working toward the same agreed goal. Early in the process, we engaged key stakeholders, holding meetings with the Peri‐Operative and Women's Health Service managers, theatre managers and clinical leads, day stay and post‐anaesthetic care staff, as well as members of the administration teams. This ensured that all members of the multi‐disciplinary team (MDT) felt ownership of the process, and were able to offer suggestions and improvements.

Three main documents were developed for the project, which together constituted our DCTLH protocol: first; a pre‐operative information package for the patient clearly outlining the DCTLH process and expectations from a patient perspective; second, an all‐staff guidance document, which covered surgical and anaesthetic approaches, a discharge criteria checklist, and a trial of void protocol; third, a standardised discharge package for the patient which included advice and pre‐formed prescriptions. Readers are welcome to contact the lead author for a copy of the complete DCTLH protocol.

A peri‐operative anaesthesia regime was developed, with a focus on early mobilisation and reduced postoperative nausea. This included: pre‐operative administration of oral paracetamol and application of a Scopoderm patch; Propofol ‘TIVA’ anaesthesia to minimise the risk of postoperative nausea; analgesic modalities including ketamine, tramadol, fentanyl, and parecoxib; multi‐antiemetic adjuncts including IV dexamethasone. Specific innovations included the sub‐peritoneal administration of local anaesthetic using a cystoscopic needle as a pelvic nerve block, which will be described and submitted at a later date for peer review.

Patient eligibility criteria followed the Sussex Protocol, which required patients to be ASA 1 or 2, of low risk for complication, have a uterus less than 16 weeks size, have access to transport and be with a responsible adult for 24 h postoperatively [3]. A specific requirement for our regional setting was that patients stayed fewer than 30 min and 50 km drive from Nelson Public Hospital for 24 h postoperatively. Five patients were offered the chance to participate in the project and had their surgeries in August and September, 2024 (Table 1). By way of comparison to these patients' characteristics, a review of the corresponding author's surgical log‐book in the year of this study found a total of 58 TLHs: 86% (*n* = 50) were younger than this cohort's oldest patient; 83% (*n* = 48) were European and 9% (*n* = 5) were Māori; 78% (*n* = 45) were ASA 1 or 2; 78% (*n* = 45) were the same BMI or less than this cohort's highest BMI.

**TABLE 1:** Patient characteristics.

Patient	A	B	C	D	E
Age	Late 50s	Early 30s	Mid 40s	Mid 40s	Early 40s
Ethnicity	European	Māori	European	European	European
ASA	2	2	2	2	2
BMI (kg/m^2^)	39	22	27	24	18

Abbreviations: ASA, American Society of Anaesthesiologists classification; BMI, body mass index.

Patients were consulted in clinics preoperatively by both the surgeon and anaesthetist, where DCTLH was discussed, informed consents were obtained, and written information was provided. An effort was made throughout this process to provide consistent information, maintaining patient motivation and preparing them for same‐day discharge.

Postoperative follow‐up included a phone call from the surgeon the evening of the surgery and the following morning. All patients were phoned at approximately 2 weeks postoperatively for a check‐in and to confirm histology results, and then seen in clinic at 4 weeks postoperatively for a physical review. In addition, all patients were provided with their surgeon's phone‐number for access while recovering in case of issues or emergencies; one patient reached out at 1 week postoperatively with constipation.

A retrospective analysis was performed at the 4‐week postoperative review of patient demographics, operative details. Voluntary patient questionnaires were also administered. The primary outcomes were defined as successful discharge on the day of surgery, and patient acceptability of the DCTLH process assessed using a 5‐point Likert scale; this mirrored the approach of the Sussex trial to allow direct comparison.

New Zealand Health and Disability Ethics Commission was consulted, and the study was found to be outside of scope and not requiring approval. All five patients agreed to complete the voluntary patient questionnaire and have shared their answers and reflections with written consent. All have read this article in its final draft form, providing additional written consent prior to submission. Anonymisation of patient data has followed the British Medical Journal standards [5].

## 3 Results

All patients discharged home successfully on day of surgery. One patient required discharge with an indwelling urinary catheter, and returned for a successful trial of void at Day‐3 postoperatively. Additional information was collected regarding the specific details of the operations, included below (Table 2). This cohort included significant ranges of BMIs and co‐morbidities, as well as intra‐operative pathology including significant adhesions and severe endometriosis.

**TABLE 2:** Particulars of surgery and post operative evaluation statements.

Patient	A	B	C	D	E
1° Operation	TLH	TLH	TLH	TLH	TLH
2° Operation	Salpingo‐oophorectomy	Salpingectomy	Salpingo‐oophorectomy	Salpingo‐oophorectomy	Salpingectomy
3° Operation	—	Severe Adhesiolysis	Ureterolysis	Severe Adhesiolysis	—
1° Diagnosis	EIN	AUB (Fibroid)	AUB (Adenomyosis)	AUB (Adenomyosis)	AUB (Adenomyosis)
2° Diagnosis	—	—	Endometriosis Stage III	Endometriosis Stage III	Pelvic Congestion
Estimated uterine ultrasound size (cc)	91	Not performed	162	161	58
Uterine weight (g)	90	97	150	167	90
Anaesthetic time (mins)	8	8	9	8	10
Surgical time (mins)	84	46	65	94	60
Blood loss (mls)	100	200	150	200	50
PACU discharge Ready (mins)	60	115	48	77	55
Discharge same day	Yes	Yes	Yes	Yes	Yes
Total time from leaving theatre to discharge (mins)	445	453	333	498	192
Passed TROC	Yes	No	Yes	Yes	Yes
Readmission	No	No	No	No	No
I was given adequate information about the preoperative preparation	2	1	1	1	1
I was given adequate information about the recovery period	4	1	1	1	1
I would recommend a day case hysterectomy to friends and family	2	1	1	1	1

*Note:* TLH, total laparoscopic hysterectomy; EIN, endometrial intraepithelial neoplasia; AUB, abnormal uterine bleeding; TROC, trial removal of catheter; Anaesthetic time, entry to theatre to ready for surgical preparation; Surgical Time, intubated to transfer off surgical bed; PACU Discharge Ready, patient enters PACU until suitable for discharge to Day Stay Unit; 1, strongly agree; 2, agree; 3, neither agree nor disagree; 4, disagree; 5, strongly disagree.

In terms of the postoperative evaluation questions, all five patients either agreed or strongly agreed with the statement, ‘I would recommend day‐case hysterectomy to friends and family’. Four out of five participants agreed strongly with the statements ‘I was given adequate information about the preoperative preparation, and I was given adequate information about the recovery period’. One participant, disagreed with the statement ‘I was given adequate information about the recovery period’, though agreed with the other questions.

The second part of the questionnaire asked for free‐text answers regarding the patients' experience. Common themes that emerged included an appreciation for the chance to stay out of hospital; ‘It suited me, as I don't like hospitals’ (Patient A), ‘Having the option to recover in the comfort of my own home was great’ (Patient C). Many emphasised the importance of good preparation for home support; ‘It is crucial to ensure that support persons are thoroughly informed about postop care’ (Patient B), ‘Very manageable with the right help around’ (Patient C). Although one patient expressed dissatisfaction around information provided for after surgery (Patient A), others commented that they felt, ‘fully prepared’ for the procedure (Patient B). There was a high degree of patient acceptability; ‘incredible experience’ (Patient B), ‘I have been so happy with my recovery’ (Patient C), ‘[DCTLH] is an excellent option for women who require it’ (Patient D), ‘Best thing!!’ (Patient E), ‘This has completely changed my way of living—the amount of time I had to take off work was embarrassing; I felt like a let‐down. I can get back to doing the things I enjoyed doing, without horrible periods’ (Patient E).

## 4 Discussion

We have demonstrated that with MDT collaboration, patients undergoing laparoscopic hysterectomy can safely and acceptably be offered same day discharge in a regional New Zealand setting. To do this, significant multi‐stakeholder engagement was required at every stage of the patient journey.

To sustain and develop such initiatives, however, there are important barriers to cross, such as continuing to engage and educate staff, reducing bias toward admission, and promoting an expectation toward discharge. Important infrastructure and resourcing investments will be required as well, involving business cases that consider reduced inpatient stays, waiting times and cost savings. It is our hope that on this basis, system‐level conversations and changes will take place to facilitate the process of day‐case surgery more widely in hospitals such as Nelson and that support will be forthcoming from our health leaders.

We also wish to highlight that within this heterogeneous patient cohort, the central shared attribute was a commitment to the principles of enhanced recovery after surgery (ERAS), accelerating the return to baseline function and independence. As one patient noted, small changes such as walking to theatre from chairs as opposed to being wheeled to theatre on a patient bed, ‘…empowers you to feel well and not sick and ready for a procedure that once complete sees you confident to go home and recover’ (Patient D).

Since this project concluded, and with the support from hospital management, we have now established a once every 4 weeks dedicated DCTLH list at Nelson Public Hospital. We look forward to reporting these results at a later date.

## Conflicts of Interest

The authors declare no conflicts of interest.
